# Integration of mental healthcare into primary healthcare: a European perspective

**DOI:** 10.1192/j.eurpsy.2025.1656

**Published:** 2025-08-26

**Authors:** R. Wernigg, C. Phellas, C. Lionis, K. Fountoulakis, E. Kovács, P. Theodorakis

**Affiliations:** 1 Department for Primary Caree Planning and Development, National Directorate-General for Hospitals, Budapest, Hungary; 2 Medical Sociology, University of Nicosia, Nicosia, Cyprus; 3 Medical Faculty, University of Crete, Crete; 4 Medical School, Aristotle Universit of Thessalonikiy, Thessaloniki, Greece; 5 Institute of Healthcare Management, Semmelweis University; 6 Country Office Hungary, World Health Organization, Budapest, Hungary

## Abstract

**Introduction:**

The 2009 WHO and WONCA report (Integrating Mental Health in Primary Care: A Global Perspective) outlined best policies and practices for the integration of mental health into primary care. The arguments in favor are reduced stigma, improved access to care, holistic management of comorbidities, improved prevention and early detection of mental disorders, reduced losses to follow-up, lower costs, easier communication, improved social integration, protection of human rights, and improved uptake of the healthcare system (Greenhalgh T, 2009; Petersen et al, 2016). Nevertheless, mental health care in many countries remains separate from primary care, limiting access and equitable distribution. However, since the COVID-19 pandemic, the global need for mental health services have surged, causing a 25 to 27 per cent increase in the prevalence of depression and anxiety around the world, accelerating the demand for integrated care (Bower et al, 2023). So, the process of integration outlined in the 2009 WHO and WONCA report warrants an updated discussion.

**Objectives:**

International experts from Hungary, Greece, and Cyprus and of WHO, WONCA and WPA present different models of integration of mental health into primary care with special respect to public health crises.

**Methods:**

Challenges, opportunities and best practices of each country will be presented, including policy recommendations, capacity building and advocacy strategies for mental health integration and public health crises, and monitoring, evaluation, and research for integrated services.

**Results:**

In all three countries, stigma, insufficient training of primary care providers, and inadequate policy frameworks present problems. In Greece, the dominance of a medically oriented health policy has hindered interdisciplinary integration (Lionis et al., 2019). Cyprus grapples with stigma surrounding mental health issues, which affects service utilization (Nikolaou & Petkari, 2021). Opportunities are leveraging community resources and enhancing collaboration among stakeholders to foster inclusive health services (Pinaka et al., 2022). Best practices involve training programs for primary care providers, promoting awareness, and developing evidence-based policies that prioritize mental health (Ashcroft et al., 2021). Advocacy strategies should focus on engaging policymakers and the community to address mental health needs, particularly in light of public health crises like COVID-19, which have exacerbated mental health issues (Galanis et al., 2020; Maulik et al., 2020). Monitoring and evaluation are crucial for assessing the effectiveness, ensuring accountability, and adapting strategies based on research findings (Glover-Wright et al., 2023; Saxena & Kline, 2021).

**Conclusions:**

The need of updating the 2009 WHO and WONCA report is considered to include the latest evidence, experiences, and recommendations on mental health integration into primary care.

**Disclosure of Interest:**

None Declared

